# Supplementary material for: Mapping the evolution of fertility support policies in China: A content and instrumental analysis
Source: PLoS One. 2025 Oct 9;20(10):e0332137. doi: 10.1371/journal.pone.0332137 (PMC12510515; doi:10.1371/journal.pone.0332137)
Supplement: S1 Appendix — (ZIP) [file pone.0332137.s001.zip › S1 Appendix. 226 original policy documents/180-国家计划生育委员会计划生育系统宣传品管理办法(FBM-CLI-4-39450).doc]

国家计划生育委员会计划生育系统宣传品管理办法

**发布部门：** [国家计划生育委员会(已更名)](https://www.pkulaw.com/law/chl?Aggs.IssueDepartment=60314) [机构沿革](javascript:void(0))

**发文字号：**中华人民共和国国家计划生育委员会令第4号

**发布日期：**2001.05.22

**实施日期：**2001.05.22

**时效性：** [失效](https://www.pkulaw.com/law/chl?Aggs.TimelinessDic=02)

**效力级别：** [部门规章](https://www.pkulaw.com/law/chl?Aggs.EffectivenessDic=XE0301)

**法规类别：** [人口计划管理](https://www.pkulaw.com/law/chl?Aggs.Category=09701)

**失效依据：**本篇法规已被《国家人口和计划生育委员会令第11号－－人口计生委决定废止＜计划生育系统宣传品管理办法＞》（发布日期：2007年12月28日 实施日期：2007年12月28日）废止

中华人民共和国国家计划生育委员会令(第4号)

　　《国家计划生育委员会计划生育系统宣传品管理办法》，已经国家计生委委主任会议通过，现予发布施行。
　　　　　　　　　　　　　　　　　　　　　　　　　　　主　任： 张维庆
　　　　　　　　　　　　　　　　　　　　　　　　 二00一年五月二十二日
　　　　　　　国家计划生育委员会计划生育系统宣传品管理办法

第一章　总则

　　**第一条**　为了加强计划生育系统宣传品管理，规范计划生育宣传品制作和发行，保证宣传品质量，为广大群众提供计划生育优质服务，依据有关法律法规，特制定本办法。

　　**第二条**　本办法适用于计划生育系统使用的、面向群众的计划生育宣传品，包括：

　　（一）通过合法出版单位出版的、以人口和计划生育为主要内容的、计划生育系统使用的各类公开出版物。

　　（二）由新闻出版部门批准，计划生育系统组织制作，用于向群众宣传的成册、折页、散页、挂图等计划生育内部出版物。

　　**第三条**　县级及以上计划生育行政管理部门授权宣传教育职能部门行使本《办法》规定的管理职能。

第二章　宣传内容与原则

　　**第四条**　计划生育宣传品内容必须体现为人民服务的宗旨，坚持以马克思列宁主义、毛泽东思想和邓小平理论为指导，要为宣传人口与经济社会协调发展和可持续发展思想、传播计划生育和生殖保健知识、引导人们树立科学、文明、进步的婚育观念、建设社会主义生育文化服务。

　　**第五条**　计划生育宣传品的主要内容包括：

　　（一）马克思主义人口理论、新中国三代领导人关于人口与计划生育的论述、人口与经济社会协调发展和可持续发展战略；

　　（二）我国基本国情、基本国策、人口形势、人口和计划生育政策法规；

　　（三）科学、文明、进步的婚育观念，新的婚育习俗和社会风尚；

　　（四）计划生育、优生优育和生殖健康科普知识；

　　（五）人口和计划生育工作先进经验、先进典型。

　　**第六条**　计划生育宣传品的内容必须符合国家有关规定，禁止制作、传播下列内容：

　　（一）违反计划生育政策和法规的、损害计划生育工作形象的；

　　（二）不宜在民族地区宣传的计划生育内容；

　　（三）泄露国家秘密的、损害国家利益的；

　　（四）宣扬淫秽、迷信或者渲染暴力的；

　　（五）国家规定禁止出版、传播的其他内容。

　　**第七条**　有关性科学知识的宣传品，要以促进身心健康、婚姻美满、家庭幸福、社会文明为的，以科学、适度为原则，注重宣传性科学知识、性道德、性文明。

　 第三章　计划与备案

　　**第八条**　县级及以上计划生育宣教中草药心、宣传站、服务站须在上年底将次年宣传品制作计划报送同级计划生育行政管理部门；临时动议制作的宣传品须事先将制作计划报送同级计划生育行政管理部门。该部门须在１个月内报上一级计划生育行政管理部门备案。

　　**第九条**　计划生育系统制作的宣传品出版之后，制作单位除按出版管理部门规定的备案办法报送样品外，还应在１个月内向同级和上一级计划生育行政管理部门各报送两份备案样品。

　　**第十条**　县级及以上计划生育宣教中心、宣传站、服务站是计划生育系统内部出版物主要制作单位。其他单位制作计划生育内部出版物，事先须向本级计划生育行政管理部门报送制作计划，事后须报送两份备案样品。

　　第四章　宣传品制作

　　**第十一条**　计划生育宣传品内部出版物制作单位，要负责宣传品的创意、设计、撰稿、审稿、印制等工作，确保宣传品质量。

　　**第十二条**　计划生育宣传品的内容，由制作人员提出，制作单位负责审定。重要及敏感的内容须报同级计划生育行政管理部门审定。

　　**第十三条**　宣传品制作单位在制作计划生育内部出版物之前，必须按有关规定向新闻出版行政部门提出申请，获得准印许可后方可印制。

　　**第十四条**　计划生育宣传品的印制必须委托取得《出版物印制许可证》的企业印制，严格执行国家有关书刊印刷标准和印刷产品质量监督管理的规定。

　　**第十五条**　计划生育宣传品（包括公开出版物、内部出版物）的著作权，属于作者或其他依法享有著作权的公民、法人或其他组织。任何单位或个人不得以任何借口在计划生育宣传品制作中侵犯他人的著作权。

　第五章　宣传品发行

　　**第十六条**　各级计划生育行政管理部门要建立、健全计划生育宣传品发行网络，管理本系统计划生育宣传品的发行。

　　**第十七条**　提倡各省（区、市）计划生育宣教中心联合制作与发行计划生育宣传品；要优势互补、合理配置资源，避免浪费。

　　禁止对计划生育宣传品发行实行地区封锁。

　　**第十八条**　国家和省级计划生育行政管理部门按照管理权限负责对计划生育宣传品的质量和宣传效果进行评估，并组织对优秀计划生育宣传品的评选和推荐。

　　**第十九条**　国家评选出的优秀计划生育宣传品，冠以“国家推荐的优秀计划生育宣传品”，作为重点宣传品在全国计划生育系统推荐发行。

　　**第二十条**　各级计划生育行政管理部门及所属单位不得订购非法印制的计划生育宣传品。

第六章　宣传品制作经费

　　**第二十一条**　各级计划生育行政管理部门要保证制作、发放计划生育宣传品的经费，确保计划生育宣传品进村入户。

　　**第二十二条**　计划生育宣传品制作单位须按《[内部资料性出版物管理办法](https://www.pkulaw.com/chl/65b4d7517d100b2ebdfb.html)》有关规定，承担内部出版物制作经费。

　 第七章　罚则

　　**第二十三条**　违反本办法第六条规定，制作传播违反国家法律法规、有损国家利益宣传品的，计划生育行政管理部门对主要负责人给予警告、通报批评；情节严重的，提请新闻出版行政管理部门依据有关规定处理；触犯[刑法](https://www.pkulaw.com/chl/85ce290bee0eaaf9bdfb.html)，构成犯罪的，移交司法机关依法追究其刑事责任。

　　**第二十四条**　违反本办法第八条、第九条、第十条、第十七条规定，未报送制作计划或样品、对计划生育宣传品实行地区封锁的，由计划生育行政管理部门对有关单位给予警告、通报批评。对主要负责人给予行政处分。

　　**第二十五条**　违反本办法第十四条、第二十条规定的，计划生育行政管理部门对主要责任人给予行政处分。

　　**第二十六条**　违反本办法第十五条规定，侵犯著作权人合法权益，情节较轻的，计划生育行政管理部门对主要负责人给予行政处分；情节严重者，提请著作权行政管理部门依法给予处罚；触犯[刑法](https://www.pkulaw.com/chl/85ce290bee0eaaf9bdfb.html)、构成犯罪者，移交司法机关依法追究其刑事责任。

　　 第八章　附则

　　**第二十七条**　以人口与计划生育为主要内容、通过合法出版单位出版发行的、用于计划生育系统使用的各类公开出版物的制作按照有关规定办理，其发行依照本办法执行。

　　**第二十八条**　本办法由国家计划生育委员会负责解释。

　　**第二十九条**　本办法自发布之日起施行。

© 北大法宝：（[www.pkulaw.com](https://www.pkulaw.com/)) 专业提供法律信息、法学知识和法律软件领域各类解决方案。北大法宝为您提供丰富的参考资料，正式引用法规条文时请与标准文本核对。
欢迎查看所有[产品和服务](https://www.pkulaw.com/helps/2.html)。法宝快讯：[如何快速找到您需要的检索结果？](https://www.pkulaw.com/helps/70.html)[法宝](https://www.pkulaw.com/helps/69.html)V6有何新特色？

扫描二维码阅读原文

**原文链接：**<https://www.pkulaw.com/chl/7e800089a0fcb68abdfb.html>
